# Cardiac Disease in Adolescents With Delayed Diagnosis of Vertically Acquired HIV Infection

**DOI:** 10.1093/cid/cis911

**Published:** 2012-10-24

**Authors:** Robert F. Miller, Juan P. Kaski, James Hakim, Jonathan Matenga, Kusum Nathoo, Shungu Munyati, Sujal R. Desai, Elizabeth L. Corbett, Rashida A. Ferrand

**Affiliations:** 1 Research Department of Infection and Population Health, Institute of Epidemiology and Healthcare; 2 Institute of Cardiovascular Science, University College London; 3 Clinical Research Department, London School of Hygiene and Tropical Medicine; 4 Department of Cardiology, Great Ormond Street Hospital for Children; 5 Department of Radiology, King's College London, King's Health Partners, King's College Hospital NHS Foundation Trust, London, United Kingdom; 6 Department of Medicine; 7 Department of Paediatrics, University of Zimbabwe; 8 Biomedical Research and Training Institute, Harare, Zimbabwe; 9 Malawi-Liverpool Research Program, University of Malawi, Blantyre

**Keywords:** adolescent, Africa, cardiac disease, cardiomyopathy, vertically-acquired HIV

## Abstract

***Background.*** At least one-third of human immunodeficiency virus (HIV)–infected infants survive to adolescence even without antiretroviral therapy (ART), but are at high risk of complications including cardiac disease. We investigated the characteristics of cardiac disease among adolescents with HIV infection diagnosed in late childhood who were receiving ambulatory HIV care in Harare, Zimbabwe.

***Methods.*** Consecutive adolescents with vertically acquired HIV attending 2 HIV outpatient treatment clinics were studied. Assessment included clinical history and examination, and 2-dimensional, M-mode, pulsed- and continuous-wave Doppler echocardiography.

***Results.*** Of 110 participants (47% male; median age, 15 years; interquartile range, 12–17 years), 78 (71%) were taking ART. Exertional dyspnea, chest pain, palpitations, and ankle swelling were reported by 47 (43%), 43 (39%), 10 (9%), and 7 (6%), respectively. The New York Heart Association score was ≥2 in 41 participants (37%). Echocardiography showed that 74 participants (67%) had left ventricular (LV; septal and/or free wall) hypertrophy and 27 (24%) had evidence of impaired LV relaxation or restrictive LV physiology. The estimated pulmonary artery systolic pressure (ePASP) was >30 mm Hg in 4 participants (3.6%); of these 2 also had right ventricular (RV) dilatation. Another 32 participants (29%), without elevated ePASP, had isolated RV dilatation.

***Conclusions.*** A significant burden of cardiac disease was seen among adolescents with vertically acquired HIV infection. More than half were asymptomatic yet had significant echocardiographic abnormalities. These findings highlight the need to screen this population in order to better define the geography, natural history, etiopathogenic mechanisms, and management (including the timing and choice of optimal therapeutic ART and cardiac drug interventions) to prevent development and/or progression of HIV-associated cardiac disease.

## Abstract (precis)

A high burden of cardiac disease was found among 110 consecutive adolescents with vertically-acquired human immunodeficiency virus infection in Zimbabwe: 37% had an NYHA score =2:echocardiography showed two thirds had LVH, 24% had LV diastolic dysfunction and 31% had RV dilatation.

Of the current estimated 2.5 million global human immunodeficiency virus (HIV) infections among children (age 0–14 years) >90% have occurred in sub-Saharan Africa, predominantly owing to mother-to-child transmission [1]. At least one-third of HIV-infected infants have slow-progressing disease with a 50% probability of surviving to adolescence even without HIV treatment [2–4]. An estimated 3% of all 10-year-olds living in Southern Africa are estimated to be “slow progressors,” the majority undiagnosed [4, 5]. These regional estimates of the burden of HIV infection reflect the high HIV prevalence rates among pregnant women during the 1990s and the lack of interventions to prevent mother-to-child transmission at that time. These children typically present to clinicians in older childhood and early adolescence with pronounced advanced immunosuppression or with longstanding chronic complications typical of pediatric HIV/AIDS, notably lung and cardiac disease [6, 7]. We have recently described the high burden of chronic lung disease, due to previously undescribed small airway disease, among adolescents with vertically acquired HIV infection who were receiving ambulatory HIV care at 2 public sector hospitals in Harare, Zimbabwe [6], and in a previous study of hospitalized HIV-infected adolescents we also recorded cardiac disease (including dilated cardiomyopathy) in 12% [7].

Cardiac disease is well described in HIV-infected infants and young children in both high- and low-resource settings [8–16]. Frequently described abnormalities include left ventricular (LV) diastolic dysfunction, dilated cardiomyopathy, increased LV wall thickness or mass, decreased LV fractional shortening, and pericardial effusion [8–16]. By contrast, data on cardiac abnormalities among HIV-infected older children and adolescents are scarce.

The aim of the present study was to describe the clinical characteristics and echocardiographic features of cardiac disease among the previously described cohort of adolescents with vertically acquired HIV infection receiving outpatient HIV care in Harare, Zimbabwe [6].

## METHODS

### Patients

Consecutive patients aged 10 to 19 years attending the HIV outpatient clinics at Harare Central and Parirenyatawa Hospitals, Harare, were enrolled into a study to investigate lung and cardiac disease [6]. Exclusion criteria including having HIV infection that was probably horizontally acquired, residing outside of Harare, being too ill to participate (ie, needing immediate hospitalization), being pregnant, or having pulmonary Kaposi sarcoma, recently diagnosed tuberculosis, or an intercurrent acute lower respiratory tract infection. HIV infection was considered vertically acquired if the following specific criteria were present: maternal and/or sibling death or known maternal HIV infection as well as no history of sexual debut or blood transfusion, history of frequent early childhood infections, or stunting or pubertal delay, as described elsewhere [6, 17].

### Data Collection

Participant demographics, stage of HIV infection (using the World Health Organization [WHO] adult classification) [18], receipt of antiretroviral therapy (ART), and symptoms (including New York Heart Association [NYHA] functional score) were recorded on a standard proforma. All participants underwent a clinical examination including height and weight measurement, recording of heart rate, pulse oximetry (arterial oxygen saturation at rest and after exercise) and presence of ankle edema. All participants had a CD4 cell count measured (CyFlow counter; Partec) and underwent transthoracic echocardiography. The *z* scores for height and weight for age were calculated using British 1990 growth reference curves [19].

### Echocardiographic Examination

Transthoracic echocardiography was done using a Toshiba Xario machine (Toshiba Medical Systems) with a 3.0-MHz transducer. Consensus criteria for echocardiographic measurements, based on recommendations of the American Society of Echocardiography [20, 21] were developed by the 2 study cardiologists (J. H. and J. M.) in joint sessions on 10 adolescent patients (who were not subsequently included in the present study). A standard protocol was used with 2-dimensional, M-mode, pulsed- and continuous-wave Doppler echocardiography and color flow mapping. Scanning was done in the left parasternal long and short axes, apical 2-chamber, 4-chamber, and subcostal views [21]. The measurement of chamber dimensions, estimation of ejection fraction, and fractional shortening were performed according to recommendations of the American Society of Echocardiography [20]. The peak estimated pulmonary arterial systolic pressure (ePASP) was derived by calculating the systolic pressure gradient between the right ventricle and right atrium by the maximum velocity of the tricuspid regurgitant jet, using the modified Bernoulli equation, and then adding 10 mm Hg unless the right atrial pressure was judged to be high (in which case 15 mm Hg was added) or low (in which case 5 mm Hg was added) [22]. For all measurements, the final value was the average of measurements performed in 3 sequential cardiac cycles.

### Statistical Analysis

Stata software (version 10.0; StataCorp) was used for analyses. Echocardiographic parameters were expressed as a deviation from the body surface area–corrected mean (*z* scores), based on published normal values published elsewhere [23, 24]. The following definitions were used: LV dilatation, LV end-diastolic dimension *z* score >2; LV hypertrophy, maximal wall thickness (interventricular septum or LV posterior wall [LVPW]) *z* score >2; “concentric” LV hypertrophy, both interventricular septum and LVPW *z* scores >2; left atrial (LA) dilatation, LA *z* score >2; LV systolic impairment, fractional shortening <25% or ejection fraction <50%; LV diastolic dysfunction, mitral inflow E/A ratio (the ratio between peak early [E] and late diastolic [A] transmitral ventricular filling velocities) <1, or >2; right ventricular (RV) dilatation, RV end-diastolic dimension *z* score >2; and pulmonary hypertension, ePASP >30 mm Hg.

The association between clinical variables (sex, age, height for age, weight for age, and body mass index, WHO stage, CD4 cell count, and duration and type of ART [zidovudine and/or a protease inhibitor–containing regimen]) and specific echocardiographic abnormalities (LV hypertrophy, LV diastolic dysfunction, and RV dilatation) were investigated using the χ^2^ test for categorical variables and the Mann-Whitney *U* test for variables that were not normally distributed.

Variables that showed an association at a significance level of *P* < .1 were analyzed using multivariate logistic regression analysis; differences were considered significant at *P* < .05.

### Ethics

All participants gave their assent to participate in the study. Written informed consent in either English or Shona was also obtained from the guardians of all participants. Ethical approval was obtained from the Medical Research Council of Zimbabwe, the Biomedical Research and Training Institute Institutional Review Board, Harare, and the London School of Hygiene and Tropical Medicine Ethics Committee.

## RESULTS

### Baseline Clinical Characteristics

Baseline demographic and clinical characteristics of 110 participants (median age, 15 years; interquartile range [IQR], 12–17 years; 47% male) are shown in Table 1. Seventy-eight (70.9%) were taking ART, for a median duration of 20 months (IQR, 5–40 months). The median CD4 cell counts did not differ between those taking ART and those who were not (402 [IQR, 196–590] vs 351 [77–535] cells/µL; *P* = 0.16). Of those receiving ART, the regimen included zidovudine in 13, of whom 6 also received a protease inhibitor.

**Table 1.** Baseline Demographic and Clinical Characteristics Among 110 Adolescents With Vertically Acquired Human Immunodeficiency Virus Infection

Characteristic	No. (%)
Age, y	
<13	28 (25.5)
13–15	30 (27.2)
16–19	52 (47.3)
Male sex	52 (47)
Height-for-age *z* score, median (IQR)	−2.22 (−3.05 to −1.3)
Weight-for-age *z* score, median (IQR)	−1.84 (−3 to −0.94)
BMI *z* score, median (IQR)	−0.69 (−1.81 to 0.11)
Taking ART	78 (71)
Regimen	
2 NRTIs + 1 NNRTI	70 (63.6)
2 NRTIs + PI	6 (5.5)
Not known	2 (1.9)
CD4 count, median (IQR), cells/µL	384 (171–578)
Symptoms and signs	
Shortness of breath on exertion	47 (43)
Chest pain on exertion	43 (39)
Palpitations	10 (9)
Ankle swelling	7 (6)
NYHA functional classification	
I	69 (63)
II	18 (16)
III	21 (20)
IV	2 (2)
Tachycardia at rest (HR >100/min)	31(28)
SaO_2_ at rest <92%	16 (14.5)

Abbreviations: ART, antiretroviral therapy; BMI, body mass index; HR, heart rate; IQR, interquartile range; NNRTI, nonnucleoside reverse-transcriptase inhibitor; NRTI, nucleoside reverse-transcriptase inhibitor; NYHA, New York Heart Association; PI, protease inhibitor; SaO_2_, arterial oxygen saturation.

No participants were cigarette smokers, and none were receiving angiotensin-converting enzyme inhibitors, β-blockers, calcium channel blockers, digoxin, diuretics, or vasodilators. Sickle cell disease and congenital and rheumatic heart disease were excluded in all participants: 1 had a previous diagnosis of dilated cardiomyopathy. Forty-one participants (37%) were symptomatic (NYHA score ≥2), and at rest 31 (28%) were tachycardic (heart rate >100 beats per minute) and 16 (14.5%) were hypoxemic (arterial oxygen saturation <92%).

### Echocardiographic Findings

**Figure 1.**
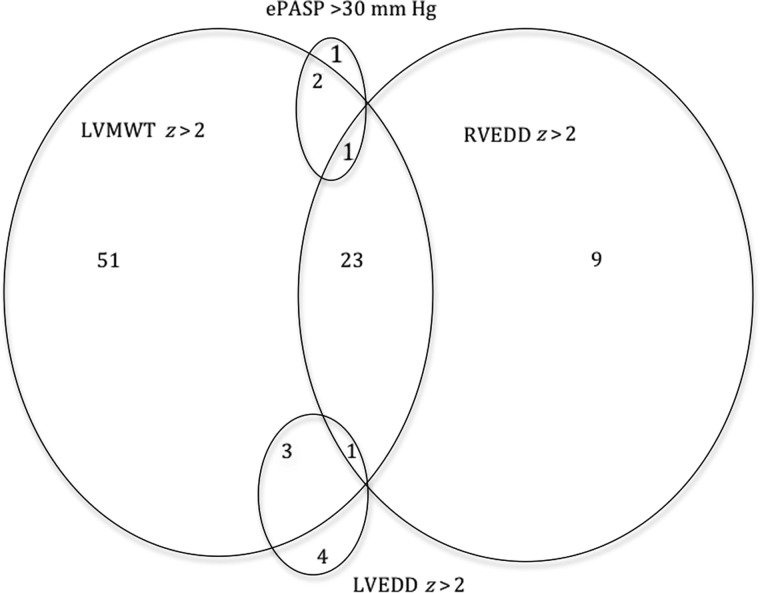
Prevalence of echocardiographic findings among 110 adolescents with vertically acquired human immunodeficiency virus infection. Unless otherwise specified, numbers represent numbers of adolescents with finding. Abbreviations: ePASP, estimated pulmonary artery systolic pressure; LVEDD, left ventricular end-diastolic dimension; LVMWT, left ventricular maximum wall thickness; RVEDD, right ventricular end-diastolic dimension; *z, z* score.

Echocardiographic findings in the 110 participants are summarized in Figure 1 and Table 2.

**Table 2.** Echocardiographic Characteristics Among 110 Adolescents With Vertically Acquired HIV Infection

Measurement	Result, Median (IQR)	z Score, Median (IQR)
RVEDD, mm	15.0 (12.6–17.5)	+1.14 (+0.09 to 2.17)
LVEDD, mm	38.7 (34.5–42.5)	−0.48 (−1.47 to 0.34)
LVESD, mm	24.1 (21.3–26.9)	−0.81 (−1.44 to 0.18)
IVS, mm	8.1 (6.7–9.6)	+1.89 (+0.64 to 3.39)
LVPW, mm	8.7 (7.1–9.9)	+1.82 (+0.69 to 2.62)
LA, mm	25.3 (22.0–28.3)	−0.18 (−1.39 to 0.80)
FS, %	38.7 (31.5–42.9)	…
EF, %	69.3 (60.1–74.8)	…
ePASP, mm Hg	12.0 (11.3–15.2)	…
E/A ratio	1.58 (1.36–1.93)	…
LV mass, g	85.7 (72.5–116.3)	−0.14 (−1.06 to 0.87)

Abbreviations: E/A ratio, ratio between peak early (E) and late diastolic (A) transmitral ventricular filling velocities; EF, ejection fraction; ePASP, estimated pulmonary artery systolic pressure; FS, fractional shortening; IQR, interquartile range; IVS, interventricular septum; LA, left atrial dimension; LV, left ventricular; LVEDD, LV end-diastolic dimension; LVESD, LV end-systolic dimension; LVPW, LV posterior wall; RVEDD, right ventricular end-diastolic dimension.

#### LV Dilatation and Systolic Impairment

**Figure 2.**
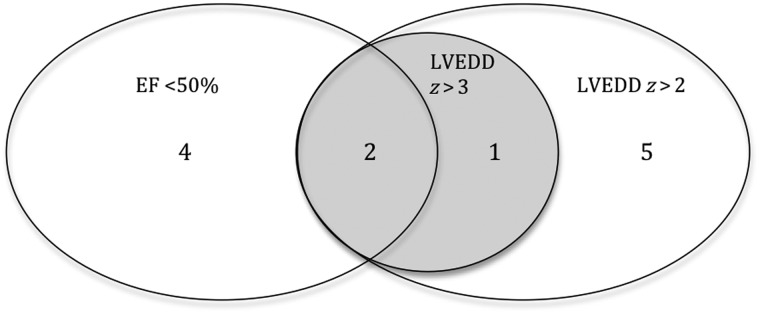
Prevalence of left ventricular dilatation and systolic impairment among 110 adolescents with vertically acquired human immunodeficiency virus infection. Unless otherwise specified, numbers represent numbers of adolescents with finding. Abbreviations: EF, ejection fraction; LVEDD, left ventricular end-diastolic dimension; *z, z* score.

In total, 12 participants (10.9%) had LV dilatation (n = 8 [7.3%]) and/or systolic impairment (n = 6 [5.5%]; Table 2). Of these, 3 participants (2.7%) had LV end-diastolic dimension *z* scores >3, and 3 (2.7%) with LV dilatation also had mild mitral valve regurgitation (Figure 2). Seven participants (6.4%) had a LV mass *z* score >2, including the 2 with both LV dilatation and systolic impairment.

#### LV Hypertrophy

A total of 74 participants (67.2%) had LV hypertrophy, with a septal wall thickness *z* score >2 (n = 53 [48.1%]) or LVPW thickness *z* score >2 (n = 48 [43.6%]); 27 (24.5%) had both septal and LVPW thickness *z* scores >2 (“concentric” LV hypertrophy). Using a more conservative definition of LV hypertrophy (LV maximal wall thickness *z* score >3), 48 (43.6%) of participants had LV hypertrophy (septal wall thickness *z* score >3, n = 35 [31.8%]; LVPW thickness *z* score >3, n = 17 [15.5%]), including 7 (6.4%) with concentric LV hypertrophy.

#### LV Diastolic Dysfunction

Four participants (3.6%) had a mitral inflow E/A ratio <1, implying impaired LV relaxation, whereas 23 (20.9%) had E/A ratios >2, implying restrictive LV physiology. Of these 23 participants, 3 had concomitant LA dilatation. Another 5 participants had LA dilatation with a normal mitral E/A ratio; 2 had concomitant LV dilatation, and the remaining 3 had evidence of LV hypertrophy.

#### Pulmonary Hypertension

A tricuspid valve regurgitant jet was present in all individuals, allowing Doppler-derived ePASP to be calculated. Eight participants (7.3%) had an ePASP >25 mm Hg. Of these, 4 (3.6%) had an ePASP >30 mm Hg. In 2 (1.8%), this was associated with RV dilatation, including 1 individual with concomitant LV dilatation.

#### RV Dilatation

Another 32 individuals (29%), without an elevated ePASP, had isolated RV dilatation, including 10 (9.1%) with an RV end-diastolic dimension *z* score >3.

#### Other Abnormalities

None of the 110 individuals had intracavitary thrombus, pericardial thickening, or an effusion.

### Clinical Correlates of Cardiac Dysfunction

In univariate analysis, female sex, stunting (height-for-age *z* score >2), and CD4 cell count were associated with LV diastolic dysfunction. Participants' body mass index, CD4 cell count, and duration of ART were associated with LV hypertrophy, and duration of ART was associated with RV dilatation. At multivariate analysis, only female sex and stunting remained significantly associated with LV diastolic dysfunction, and duration of ART remained significantly associated with RV dilatation; no significant associations remained for LV hypertrophy. Multivariate analysis showed that female study participants and those with stunting were more likely to have LV diastolic dysfunction; (odds ratio, 2.86 [95% confidence interval, 1.05–7.69] and 3.70 [1.41–10.0], respectively. RV dilatation was associated with duration of ART, those with a >12-month exposure to ART had a higher odds of RV dilatation than those taking ART for a shorter period (odds ratio, 3.48; 95% confidence interval, 1.04–11.65). Of note, there was no association between any echocardiographic abnormality and participants' age, weight for age (wasting), body mass index, WHO stage, CD4 cell count, NYHA classification, or receipt of a zidovudine- or protease inhibitor–containing regimen of ART.

## DISCUSSION

The major finding of this study was the significant burden of cardiac disease among HIV-infected adolescents with vertically acquired HIV infection receiving ambulatory HIV care. Echocardiography showed that more than two-thirds of participants had LV hypertrophy, with 24% having impaired LV diastolic dysfunction and 4 participants (3.6%) having an elevated ePASP: furthermore, 29% of participants without an elevated ePASP had RV dilatation. Of note, more than half of participants were asymptomatic despite the high frequency of echocardiographic abnormalities. The finding of LV hypertrophy should be interpreted with caution, because the *z* scores were based on a predominantly white European population and so may be overreported [23]. In addition, wall thickness measurements were made using M-mode, which has a lower spatial resolution than 2-dimensional imaging, and may therefore overestimate the degree of hypertrophy. However, even when a more rigorous cutoff of *z* score >3 was used, >40% of participants had LV hypertrophy.

The surprising finding in the present study was that almost a third of participants had RV dilatation, in the absence of an elevated ePASP. The finding of RV dilatation and/or dysfunction in the present study may be a consequence of chronic lung disease described elsewhere among this cohort of participants; 45% of whom had an forced expiratory volume in 1 second <80% and 47% an abnormal chest radiograph. The most likely cause was small airway disease, probably constrictive obliterative bronchiolitis, with or without bronchiectasis [6].

RV hypertrophy and dilatation, ascribed to cor pulmonale, has previously been reported in 48% of symptomatic HIV-infected children (median age, 9 months) in Zimbabwe [25]. RV dilatation has also been described in HIV-infected children and adolescents in studies from both Africa and the United States [14, 26]; in a pediatric study from Uganda, participants had a mean age of 6.8 years, and RV dilatation was more common among those with advanced HIV disease [14]. Neither of these studies describe coexisting pulmonary disease [14, 26]. The prospective, longitudinal P^2^C^2^ HIV study does not specifically describe RV size or function [8–12]. Alternatively, it might be postulated that HIV-infected individuals in this geographic setting have either an associated underlying genetic predisposition to dilated cardiomyopathy [27, 28] or that cofactors might be important, such as cytomegalovirus, coxsackie, adenovirus, or parvovirus infection [29] or dietary factors, including selenium deficiency [30]. These potential cofactors were not assessed in the present study.

Our study found no evidence of associations between echocardiographic evidence of LV hypertrophy, LV diastolic dysfunction, and RV dilatation and many clinical variables, including participants' age, “wasting,” body mass index, NYHA classification, CD4 cell count, or receipt of an zidovudine- and/or protease inhibitor–containing regimen of ART. However, LV diastolic dysfunction seemed to be associated with female sex and with stunting, and RV dilatation with receipt (but not type) of ART for ≥12 months. These findings are similar to those from a study of HIV-infected children (median age, 36 months) from Thailand, which found that echocardiographic evidence of LV diastolic dysfunction did not correlate with participants' age, clinical symptoms and signs, nutritional status, or HIV stage [16]. By contrast another study of HIV-infected children (mean age, 6.8 years), performed in Kampala, Uganda, showed HIV stage was associated with echocardiographic evidence of LV diastolic dysfunction, but not with LV hypertrophy or RV dilatation: other clinical variables, including wasting, stunting, NYHA classification, and participants' receipt of ART, were not recorded [14].

Another study from Lagos, Nigeria, showed no correlation between echocardiographically determined LV hypertrophy and LV systolic and diastolic dysfunction among HIV-infected children (median age, 44.2 months) and participants' age, sex, or HIV stage; however these echocardiographic abnormalities were associated with receipt of ART [15]. Furthermore, in a study of HIV-infected children (mean age, 2.8 years) in Boston, Massachusetts, LV mass was reported to have an inverse relationship with weight and height *z* scores and arm muscle circumference [31]. The inconstant associations between clinical variables and echocardiographic abnormalities in these studies and in the present study probably in part reflect differences in the demographics of participants and in study design.

The underlying mechanism by which HIV induces cardiac disease remains unclear. In a rodent model, the HIV protein gp120 has been shown to exert a direct negative inotropic effect on ventricular myocytes [32,] resulting in a selective defect in the diastolic relaxation response to adrenergic stimulation [33]. Defective adrenergic signaling is common to both ischemic and nonischemic cardiomyopathy [34]. Most reports describe HIV RNA and viral proteins predominantly in cardiac interstitial macrophages and lymphocytes, rather than in myocytes; however, a strong correlation between the extent of simian immunodeficiency virus replication in myocardium and diastolic dysfunction has been described in macaques [35]. These data imply a direct pathogenic role for HIV or viral proteins in causing myocardial dysfunction and suggest that early intervention with ART may prevent HIV-cardiac myocyte interaction, thus limiting myocardial HIV replication and subsequent myocardial damage and preventing the subsequent development of HIV-associated cardiac disease [36].

Earlier diagnosis of HIV infection may be important for preventing the development of symptomatic cardiac disease and international recommendations, which currently do not support immediately starting ART in children with HIV infection diagnosed at age ≥2 years, need to take into account the risk of developing cardiac disease and the potential for early initiation of ART to prevent or to slow its progression [37]. Against this is evidence from the present study and another study from Nigeria [15], which suggests that ART exposure might increase the risk of cardiac disease [15]. These observations are consistent with reports of an association between LV diastolic dysfunction and receipt of zidovudine [38], and between both LV diastolic dysfunction and LV hypertrophy and the duration of protease inhibitor–containing ART regimens among HIV-infected adults [39], but they contrast with a previous report from Uganda, wherein only 1 of 220 children (21% aged 13–18 years) receiving a zidovudine-containing ART regimen developed dilated cardiomyopathy [40].

The strengths of this study are its prospective design, unselected patient recruitment, the exclusion of participants who were acutely unwell, and the systematic use of 2-dimensional, pulsed- and continuous-wave Doppler echocardiography. Limitations include the cross-sectional study design, the lack of local population reference values for determining echocardiographic *z* scores, necessitating use of US and European pediatric population-derived normal values, and the lack of follow-up to correlate echocardiographic findings and their associations with morbidity and mortality, as demonstrated in other studies of HIV-infected infants and children [8, 10]. Furthermore, diastolic function was assessed only by using mitral inflow Doppler velocities; further studies using pulmonary venous Doppler and tissue Doppler imaging would provide a more accurate evaluation of diastolic abnormalities in this population.

In conclusion, this study showed a significant burden of cardiac disease among adolescents with vertically acquired HIV infection, almost three-quarters of whom were receiving ART. Echocardiographic abnormalities occurred in both symptomatic and asymptomatic individuals. More than two-thirds had echocardiographic evidence of LV hypertrophy, and a quarter had impaired LV diastolic dysfunction; RV dilatation, probably secondary to chronic lung disease, was present in more than a third. Further investigation of the geographic distribution, the role of genetic, infectious, and dietary factors in pathogenesis, the natural history, and the histopathological correlates of cardiac disease among HIV-infected adolescents in this setting is warranted to focus healthcare resources and to identify appropriate preventative and treatment interventions.

## References

[1] http://www.unaids.org/documents/20101123_GlobalReport_Annexes1_em.pdf.

[2] Marson M, Zaba B, Salomon JA, Brahmbhatt H, Bagenda D (2005). Estimating the net effect of HIV on child mortality in African populations affected by generalized HIV epidemics. J Acquir Immune Defic Syndr.

[3] Stover J, Walker N, Grassly NC, Marston M (2006). Projecting the demographic impact of AIDS and the number of people in need of treatment: updates to the Spectrum projection package. Sex Trans Infect.

[4] Ferrand RA, Corbett EL, Wood R (2009). AIDS among older children and adolescents in Southern Africa: projecting the time course and magnitude of the epidemic. AIDS.

[5] Ferrand RA, Munaiwa L, Matsekete J (2010). Undiagnosed HIV infection among adolescents seeking primary care in Zimbabwe. Clin Infect Dis.

[6] Ferrand RA, Desai SR, Hopkins C (2012). Chronic lung disease in adolescents with delayed diagnosis of vertically-acquired HIV infection. Clin Infect Dis.

[7] Ferrand RA, Bandason T, Musivaire P (2010). Causes of acute hospitalization in adolescence: burden and spectrum of HIV-related morbidity in a country with an early-onset and severe HIV epidemic: a prospective survey. PLoS Med.

[8] Lipshultz SE, Easley KA, Orav EJ, for the Pediatric Pulmonary and Cardiac Complications of Vertically Transmitted HIV infection (P^2^C^2^ HIV) Study Group (1998). Left ventricular structure and function in children infected with human immunodeficiency virus. The prospective P2C2 HIV Multicenter Study. Circulation.

[9] Starc TJ, Lipshultz SE, Kaplan S, for the Pediatric Pulmonary and Cardiac Complications of Vertically Transmitted HIV infection (P^2^C^2^ HIV) Study Group (1999). Cardiac complications in children with human immunodeficiency virus infection. Pediatrics.

[10] Lipshultz SE, Easley KA, Orav EJ (2000). Cardiac dysfunction and mortality in HIV-infected children: the prospective P^2^C^2^ HIV Multicenter Study. Circulation.

[11] Starc TJ, Lipshultz SE, Easley KA, for the Pediatric Pulmonary and Cardiac Complications of Vertically Transmitted HIV infection (P^2^C^2^ HIV) Study Group (2002). Incidence of cardiac abnormalities in children with human immunodeficiency virus infection: the prospective P^2^C^2^ HIV study. J Pediatr.

[12] Fisher SD, Easley KA, Orav EJ, for the Pediatric Pulmonary and Cardiac Complications of Vertically Transmitted HIV infection (P^2^C^2^ HIV) Study Group (2005). Mild dilated cardiomyopathy and increased left ventricular mass predict mortality: the prospective P^2^C^2^ HIV Multicenter Study. Am Heart J.

[13] Brown SC, Schoeman CJ, Bester CJ (2005). Cardiac findings in children admitted to a hospital general ward in South Africa: a comparison of HIV-infected and uninfected children. Cardiovasc J South Afr.

[14] Lubega S, Zirembuzi GW, Lwabi P (2005). Heart diseases among children with HIV/AIDS attending the pediatric infectious disease clinic at Mulago hospital. Afr Health Sci.

[15] Okoromah CA, Ojo OO, Ogunkunle OO (2012). Cardiovascular dysfunction in HIV-infected children in a sub-Saharan African country: comparative cross-sectional observational study. J Trop Pediatr.

[16] Pongprot Y, Sittiwangkul R, Silvilairat S, Sirisanthana V (2004). Cardiac manifestations in HIV-infected Thai children. Ann Trop Paediatr.

[17] Ferrand RA, Luethy R, Bwakura F, Mujuru H, Miller RF, Corbett EL (2007). HIV infection presenting in older children and adolescents: a case series from Harare, Zimbabwe. Clin Infect Dis.

[18] World Health Organization (2005). Interim WHO clinical staging of HIV/AIDS and HIV/AIDS case definitions for surveillance—African Region.

[19] Cole TJ (1997). Growth monitoring with the British 1990 growth reference. Arch Dis Child.

[20] Gottdiener JS, Bednarz J, Devereux R (2004). American Society of Echocardiography recommendations for use of echocardiography in clinical trials. J Am Soc Echocardiogr.

[21] Henry WL, DeMaria A, Gramiak R (1980). Report of the American society of echocardiography committee on nomenclature and standards in two-dimensional echocardiography. Circulation.

[22] Kircher BJ, Himelman RB, Schiller NB (1990). Noninvasive estimation of right atrial pressure from the inspiratory collapse of the inferior vena cava. Am J Cardiol.

[23] Foster BJ, Mackie AS, Mitsnefes M, Ali H, Mamber S, Colan SD (2008). A novel method of expressing left ventricular mass relative to body size in children. Circulation.

[24] Kampmann C, Wiethoff CM, Wenzel A (2000). Normal values of M mode echocardiographic measurements of more than 2000 healthy infants and children in central Europe. Heart.

[25] Bannerman C, Chitskie I (1995). Cor pulmonale in children with human immunodeficiency virus infection. Ann Trop Paediatr.

[26] Kavanaugh-McHugh A, Ruff A, Rowe S, Holt E, Wilfert C, Modlin JF (1991). Cardiac abnormalities in a multi-center interventional study of children with symptomatic HIV infection. The ACTG 043. Pediatr Res.

[27] Herman DS, Lam L, Taylor MR (2012). Truncations of titin causing dilated cardiomyopathy. N Engl J Med.

[28] Friedrich FW, Carrier L (2012). Genetics of hypertrophic and dilated cardiomyopathy. Curr Pharm Biotechnol.

[29] Kindermann I, Barth C, Mahfoud F (2012). Update on myocarditis. J Am Coll Cardiol.

[30] Twagirumukiza M, Nkeramihigo E, Seminega B, Gasakure E, Boccara F, Barbaro G (2007). Prevalence of dilated cardiomyopathy in HIV-infected African patients not receiving HAART: a multicenter, observational, prospective, cohort study in Rwanda. Curr HIV Res.

[31] Miller TL, Orav EJ, Colan SD, Lipshultz SE (1997). Nutritional status and cardiac mass and function in children infected with the human immunodeficiency virus. Am J Clin Nutr.

[32] Kan H, Xie Z, Finkel MS (2004). P38 MAP kinase-mediated negative inotropic effect of HIV gp120 on cardiac myocytes. Am J Physiol Cell Physiol.

[33] Berzingi C, Chen F, Finkel MS (2009). P38 MAP kinase inhibitor prevents diastolic dysfunction in rats following HIV gp120 injection in vivo. Cardiovasc Toxicol.

[34] Liggett SB (2001). β-adrenergic receptors in the failing heart: the good, the bad, and the unknown. J Clin Invest.

[35] Kelly KM, Tarwater PM, Karper JM (2012). Diastolic dysfunction is associated with myocardial viral load in simian immunodeficiency virus-infected macaques. AIDS.

[36] Chen F, Bhardwaj R, Finkel MS (2012). Diastolic dysfunction following HIV infection. AIDS.

[37] World Health Organization Antiretroviral therapy for HIV infection in infants and children: towards universal access.

[38] Luo L, Ye Y, Liu Z (2010). Assessment of cardiac diastolic dysfunction in HIV-infected people without cardiovascular symptoms in China. Int J STD & AIDS.

[39] Meng Q, Lima JAC, Lai H (2002). Use of HIV protease inhibitors is associated with left ventricular morphologic changes and diastolic dysfunction. J Acquir Immune Defic Syndr.

[40] Tukei VJ, Asiimwe A, Maganda A (2012). Safety and tolerability of antiretroviral therapy among HIV-infected children and adolescents in Uganda. J Acquir Immune Defic Syndr.

